# An Investigation into the Kinetics and Mechanism of the Removal of Cyanobacteria by Extract of *Ephedra equisetina* Root

**DOI:** 10.1371/journal.pone.0042285

**Published:** 2012-08-01

**Authors:** Rong Yan, Hongli Ji, Yonghong Wu, Philip G. Kerr, Yanming Fang, Linzhang Yang

**Affiliations:** 1 College of Forest Resource and Environment Science, Nanjing Forestry University, Nanjing, China; 2 State Key Laboratory of Soil and Sustainable Agriculture, Institute of Soil Science, Chinese Academy of Sciences, Nanjing, China; 3 School of Biomedical Sciences, Charles Sturt University, Wagga Wagga, New South Wales, Australia; University of New South Wales, Australia

## Abstract

An aqueous extract of *Ephedra equisetina* root was found to induce cyanobacterial cell death. The extract displayed no negative effects on the fish populations but instead, improved the habitat conditions for the growth of macrophytes, zooplankton and bacteria because the inhibiting effects of the extracts on cyanobacteria helped clear up the water column. The removal kinetics of cyanobacteria by *E. equisetina* extract appears to be a first order process with the rate constant being extract-dose-dependent. Compounds including the flavonoids found in *E. equisetina* root kill the cyanobacteria *in vitro* at a dose of 5.0 µg extract per 100 mL water or above. The extract constituents act to disrupt the thylakoid membrane, interrupt the electronic transport, decrease the effective quantum yield, and eventually lead to the failure of photosynthesis in *Microcystis aeruginosa*. This study presents an easily-deployed, natural and promising approach for controlling cyanobacterial blooms as an emergency measure, and also provides insight into the dynamics and mechanism of the extract consisting of multiple compounds synergistically removing algae.

## Introduction

Harmful algal blooms (HABs) such as those of cyanobacteria, have significant adverse impacts on flora and fauna as well as aquatic ecosystems [1]. The main impacts are i) production of hepatotoxins which cause mortalities in fish, seabirds and mammals, ii) human illness or death via bioaccumulation of algal toxins in the food web, iii) physical damage such as the disruption of epithelial gill tissues in fish [2], and iv) oxygen depletion of the water column from bacterial cellular respiration and degradation [3].

Due to their negative impacts, several methods of preventing HABs have been proposed. These include reducing nutrient inputs to prevent eutrophication, and optimizing hydrophysical conditions to favor beneficial phytoplankton species growth [4]. Where prevention of HABs has met with limited success, additional physical and chemical remediation techniques, such as dilution, flocculation, adsorption, hydrogen peroxide (H_2_O_2_) and bio-filtration (i.e., filter-feeding fish) may offer alternative or additional bloom control [3], [4], [5]. Among the natural technologies proposed, the use of active ingredients extracted from materials such as straw is considered desirable due to their facile biodegradation and low cost of materials and operations [4], [5]. However, these procedures are not without drawbacks. For example, the application of straw has side effects including oxygen-depletion and color leaching from rotting straw [6]. Therefore, it is essential to consider simultaneously, the control of HABs and the benefit to aquatic ecosystem health when such materials are utilized.

*M. aeruginosa* is the most widely distributed cyanobacteria causing HABs in surface water worldwide [7]. It naturally produces toxins such as microcystin-AR, microcystin-LR, microcystin-RR, etc. [5]. The root of *Ephedra equisetina* (Mongolian Ephedra or Ma Huang in TCM) is recognized as a ‘poisonous but safe’ material in TCM primarily due to its alkaloid content [8]. The root of *Ephedra equisetina* is often used in cooking or home décor in China.

When natural materials such as barley straw, biofilms and the common reed, *Phragmites communis* are used for removing cyanobacteria and/or algae, multiple active ingredients tend to be concurrently released (or exuded) and then work in synergy [6], [9], [10]. Therefore, regarding different active ingredients from natural materials as an ensemble – an extract, has more potential application for removing cyanobacteria in “real world” waters than a single component in isolation. Thus, in this study, the crude extract of *Ephedra equisetina* root was proposed to induce cyanobacterial death, thereby controlling cyanobacterial blooms.

To date, attention has been focused on the dynamics of removal of cyanobacteria and/or algae by a single active compound [11], [12]. For instance, the kinetics of H_2_O_2_ used to remove cyanobacteria has been demonstrated to fit an exponential decay model [11]. However, the kinetic behavior of multiple active ingredients synergistically removing algae is still poorly understood. As a result, the dosages of the isolated active ingredients, or in their native form, are hard to quantify when confronted with the variable algal bloom levels that apply in a practical environment. Therefore, it is of significance to consider the dynamics of multiple active ingredients (i.e., a crude extract) synergistically inhibiting cyanobacteria and/or algae.

Compounds extracted from natural materials have been widely applied to control HABs (e.g., cyanobacterial blooms). These include phenolic compounds; gallic acid [10], pyrogallol, ellagic acid [13] and aliphatic acids; nonanoic, cis-6-octadecenoic, and cis-9-octadecenoic acids [14]. However, the mechanisms of action for multiple active ingredients synergistically inhibiting cyanobacterial growth are not fully known. Previous studies indicate that the removal mechanism of cyanobacteria may involve interaction among proteins [15], inhibition of alkaline phosphatase, interruption of the electron transfer chain [16], oxidant damage from auto-oxidation of polyphenol [13], [14], and alteration in the gene expression of *M. aeruginosa* [17]. To date, systematic research on the whole photosynthetic process of cyanobacteria under synergistic stress of different chemicals has not been addressed.

The aims of this study were to (i) test the effect of the aqueous extract of *Ephedra equisetina* root in the control of cyanobacterial blooms in the field, (ii) to examine the effects of the application of the extract on fish growth and macrophyte and zooplankton diversities, and (iii) to explore the dynamics and mechanism/s of action/s for the inhibition of cyanobacterial growth. In this study, we have attempted to provide a promising natural bio-measure to induce cyanobacterial death, control cyanobacterial blooms and enhance the aquatic ecosystem health. By examining the kinetics of the cyanobacterial cell death by *E. equisetina* root extract, as indicated by decreasing chlorophyll-a, we postulate mechanisms for this process under the influence of multiple compounds.

## Results and Discussion

### On-site Cyanobacterial Blooms Controlled

**Figure 1:**
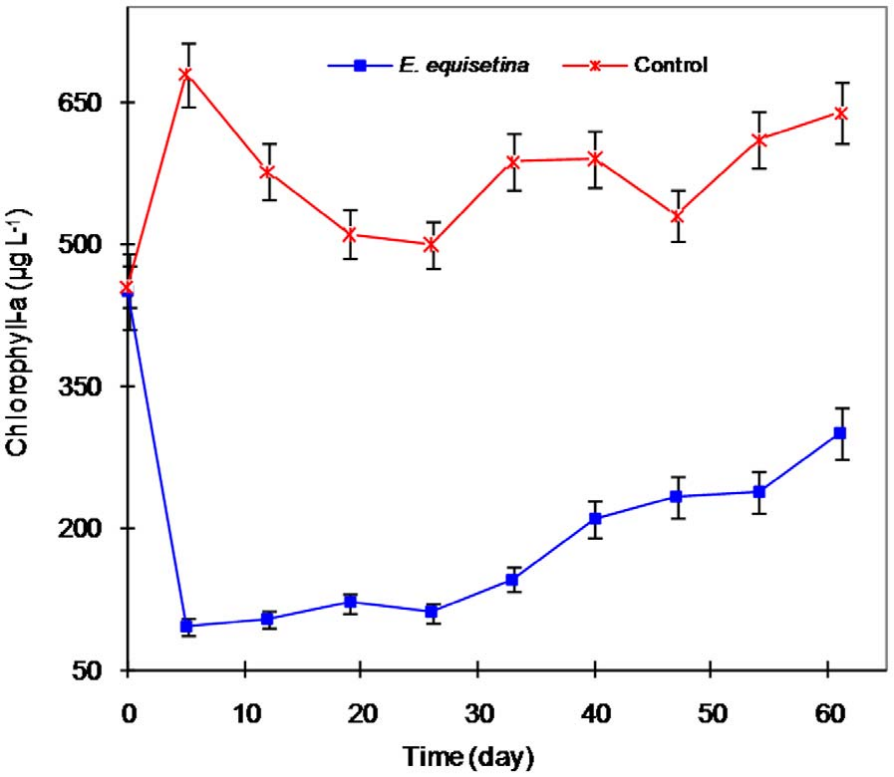
The reduction of cyanobacterial blooms using *E. equisetina* extract in an experimental versus control pond from April to June, 2008.

During the field experiment, the dominant phytoplankton in both the control ponds and the ponds treated with *E. equisetina* extracts was the cyanobacterium *M. aeruginosa*. The initial chlorophyll-a in the ponds was about 450 µg L^−1^, implying that it was the starting time of cyanobacterial blooms. The cyanobacterial population in the treated ponds was significantly reduced (*p*<0.05), as expressed by the low chlorophyll-a concentration ranging from 95 to 300 µg L^−1^ from April to June, 2008. Over the same period, the chlorophyll-a concentration in the control ponds remained at high levels, from 510 to 680 µg L^−1^ (Fig. 1). This represents heavy and frequent cyanobacterial blooms in the control ponds.

The reduced levels of chlorophyll-a in the treated ponds were observed during the whole period of cyanobacterial blooms (about 61 days). This indicates that the added dosage of extract was adequate to control cyanobacterial blooms. It also means that the change in blooms was not simply a natural restoration occurrence.

To investigate if they played a part in inhibiting cyanobacterial growth, the nutrient concentrations in the water column were determined for each pond during the experimental period (Table 1). The average TN (6.45 mg L^−1^), TP (0.65 mg L^−1^), TDP (0.23 mg L^−1^), NO_3_-N (1.77 mg L^−1^) and NH_4_-N (2.69 mg L^−1^) in the treated ponds were not significantly different from those in the control ponds (*p*>0.05). The concentrations in the treated ponds ranged from 0.11 to 0.74 mgL^−1^ (TDP), 0.44 to 3.22 mg L^−1^ (NO_3_-N) and 0.60 to 3.63 mg L^−1^ (NH_4_-N). A previous study revealed that changes in nutrient concentrations in the water column do not significantly affect the growth rate of cyanobacteria [18], [19], implying that the level of nutrients in our ponds were sufficient to support the rapid growth of cyanobacteria. Moreover, when the nutrient concentrations in the water column decrease, they might well be replenished by the release of nutrients in sediments [20]. These facts further support that the absence of cyanobacterial blooms in the experimental pond was attributable to the action of the *E. equisetina* extract.

**Table 1:** The nutrient concentrations in the control and treated (*E. equisetina* extracts) ponds during the experiment (n = 12).

Items	Range	TP	TDP	TN	NO_3_-N	NH_4_-N
Control pond	Average	0.66±0.54	0.28±0.21	6.98±1.39	2.05±0.37	2.96±0.43
	Max.	1.68	0.87	13.24	3.53	3.81
	Min.	0.43	0.10	3.68	0.82	0.67
Treated Pond	Average	0.65±0.42	0.23±0.17	6.45±1.34	1.77±0.31	2.69±0.26
	Max.	1.58	0.74	12.62	3.22	3.63
	Min.	0.34	0.11	3.52	0.44	0.60

Some chemicals such as flavonoids (proanthocyanidins), feruloylhistamine and ephedradines A–D (alkaloids) can been isolated from the roots of *Ephedra* plants such as *Ephedra sinica* [21]. Most of these compounds possess a phenol moiety and are capable of forming free radicals which can act either as anti- or pro-oxidants. Our study indicates that *E. equisetina* has robust ability to remove bacterioplankton (autotrophic bacteria) such as *M. aeruginosa*. This suggests a significant role for the cytotoxicity of the abovementioned compounds.

### Benefits to Aquatic Ecosystem

**Figure 2:**
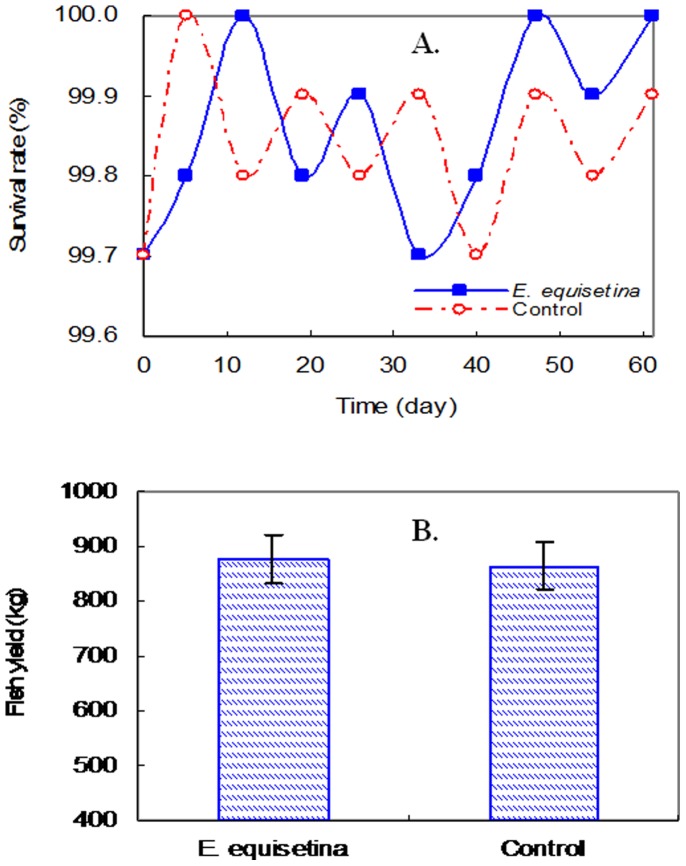
Comparison of results of the A) survival rates and B) fish yields between the pond treated with *E. equisetina* extract and the control.

To assess the effects of *E. equisetina* extract on fish, the populations of fish in the ponds were monitored. Figure 2 shows that the changes in fish survival rates and fish yields in the control ponds and in the treated ponds were not significantly different (*p*>0.05). It is indicated that the fish populations were not negatively affected by *E. equisetina* extract.

The Simpson diversity indices of macrophyte and zooplankton in the treated ponds were 2.4 and 3.6 times higher than those in the control ponds, respectively. Furthermore, the bacterial Shannon-Weaver diversity index based on flaA gene analyses in natural biofilms taken from the treated ponds was 2.3 times higher than that calculated for the control ponds (Table 2). These results suggest that there are no negative impacts on the pond ecosystems during the application of *E. equisetina* extract. They also imply that the habitat conditions for macrophytes, zooplankton, and bacteria were improved because the inhibiting effects of the extracts on cyanobacteria helped clear up the water column. In addition, the diversity indices also indicate that the aquatic ecosystems recovered to a relatively stable and healthy state after the treatment with *E. equisetina* extract.

**Table 2:** The Simpson diversity indices for macrophytes and zooplankton, and the Shannon-Weaver bacterial diversity indices (based on flaA gene) in each pond at the end of the experiment (average index ± standard error).

Items	Simpson diversity	Simpson diversity	Shannon-Weaver
Species	Macrophytes	Zooplankton	Bacteria
Control ponds	0.41±0.036	0.23±0.035	0.54±0.036
Ponds treated with *E. equisetina*	0.99±0.046	0.83±0.059	1.25±0.059

### The Kinetics of Cyanobactericidal Action

**Figure 3:**
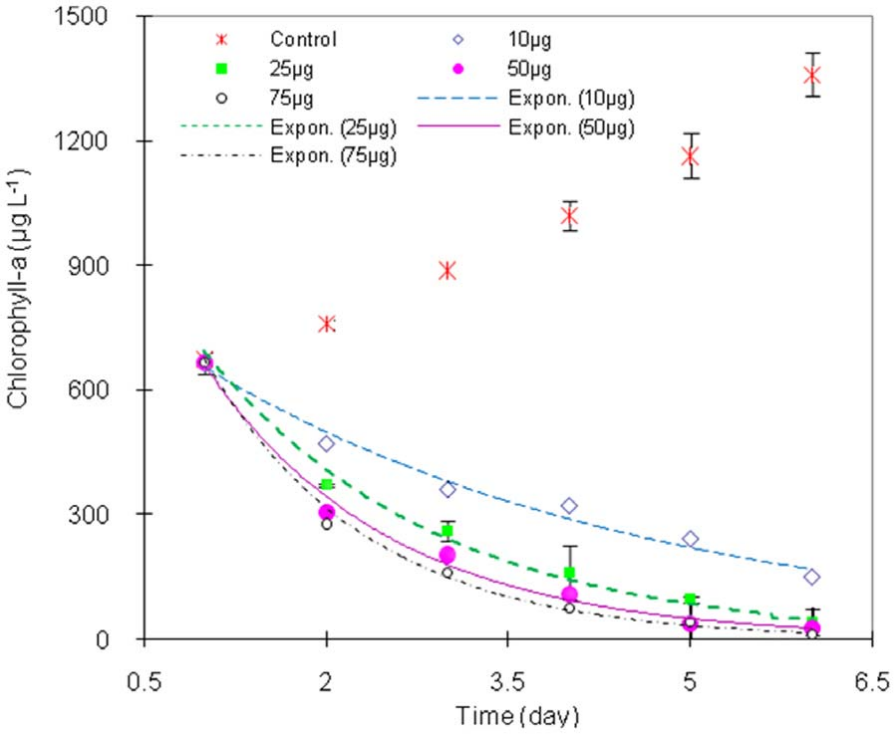
The growth of *M. aeruginosa* as expressed by chlorophyll-a concentration in the control and treatment groups of 10 µg, 25 µg, 50 µg and 75 µg aqueous *E. equisetina* extracts per 200 mL plotted as exponential regression curves against time.

The *in-vitro* bioassay results showed that while the chlorophyll-a in the controls increased rapidly from 670 µg L^−1^ to 1360 µg L^−1^ the chlorophyll-a concentration markedly decreased from 670 µg L^−1^ to 150 µg L^−1^ between the 1^st^ (t = 0) and the 6^th^ (t = 5) day when the dose of extract was 10 µg or more. Thus the treatment with *E. equisetina* extract appears to reduce viable photosynthetic *M. aeruginosa* (Fig. 3).

The data points from the samples treated with *E. equisetina* extract fit exponential decay curves with high R^2^ values (all >0.97) with statistical significance (*p*<0.01) (Table 3). This finding is strikingly similar to (but with higher R^2^ values than) the results of H_2_O_2_ at removing cyanobacteria [11], [12]. The exponential decay curves of statistical significance suggest that cyanobacterial decay exhibits first-order reaction kinetics. Mechanistically, this analysis probably indicates that the rate determining step for the ‘cell kill’, once initiated, is independent of the concentration of the plant extract.

**Table 3:** The *in vitro* kinetic parameters of *E. equisetina* extract removing cyanobacteria (*M. aeruginosa*) from eutrophic water.

Items	Dose µg	(Chl-a)_0_ µg L^−1^	*W* day^−1^	R^2^	*p*	Half-life day
*E. equisetina* extract	10	655.71	0.273	0.971	<0.01	2.54
	25	693.09	0.528	0.984	<0.01	1.31
	50	661.11	0.654	0.985	<0.01	1.06
	75	675.79	0.753	0.999	<0.01	0.92

* One-way ANOVA was performed for each treatment. *w* represents the rate constant for the applied *E. equisetina* extract dose. *p* values were determined using one-way ANOVA. Half-life  =  (ln (2))/*w*.

The reaction rate constants for cyanobacterial decay were determined from the exponential decay curves, for doses of 10, 25, 50 and 75 µg of *E. equisetina* extract (Table 3). Within this range, a plot of the rate constants versus the extract dose indicated a logarithmic dependence of cyanobacterial cell-kill on dose (R^2^ = 0.992). This implied that the removal of cyanobacteria was a more complex chemical dynamic process than a first order process. It is also likely that the decay was actually of a higher order because the “chemical attack” process was involved in multiple actions of different active ingredients [22].

### Mechanistic Considerations

**Figure 4:**
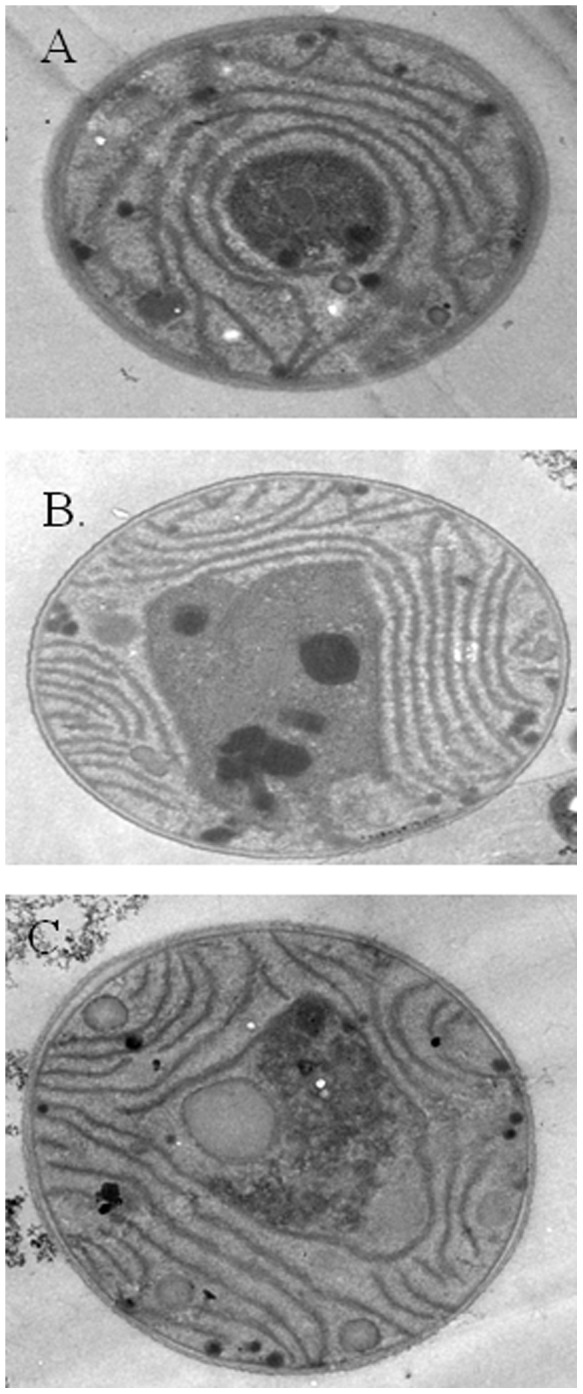
Cell structure of *M. aeruginosa* in A) the control, B)after contact with *E. equisetina* extract at a dose of 25 µg for 4 days, and C) after contact with *E. equisetina* extract at a dose of 50 µg for 6 days.

After the addition of the extract, it was observed that the dead cyanobacteria were deposited on the bottoms of the experimental flasks. We found the cell walls of *M. aeruginosa* to be intact for both control and treated samples. However, in the treated samples, the thylakoid membrane was detached from the cyanobacterial cytoplasm; the structures within the thylakoid membrane begun to deteriorate (Fig. 4). This suggested that the death of cyanobacteria was caused by “chemical attack” from the flavonoids in the extract passing through the cell membrane, and causing their damage, rather than by either “mechanical destruction” (UV exposure) or “biological competition” (other organisms).

**Figure 5:**
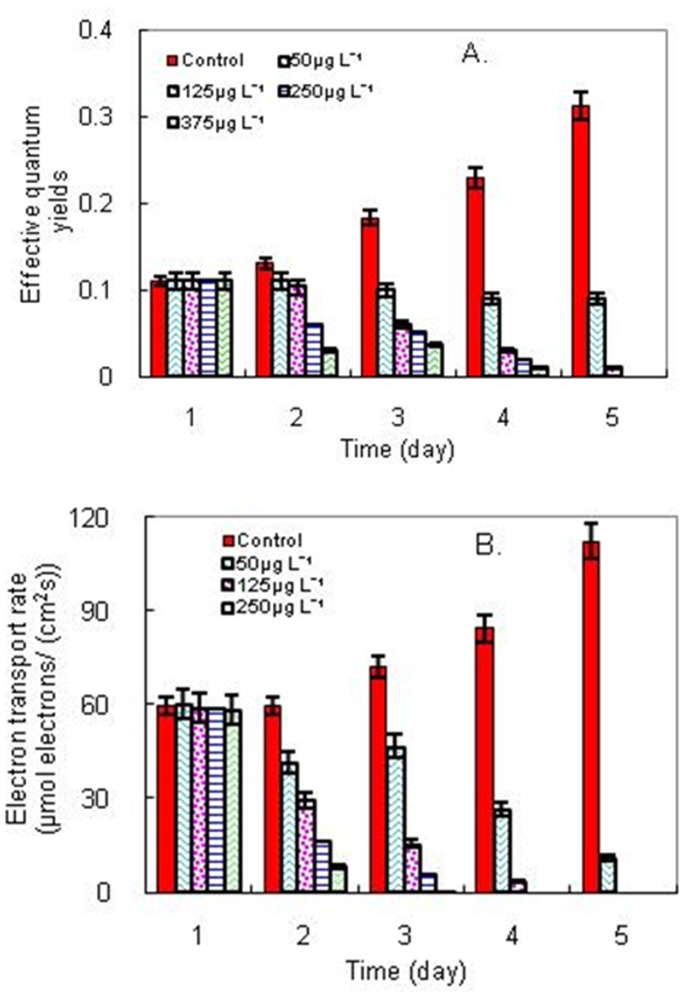
Changes of A) effective quantum yields and B) electronic transport rates in PS II reaction centers of cyanobacterial (*M. aeruginosa*) cells when exposed to *E. equisetina* extracts.

As the thylakoid membrane is the site of the light-dependent reactions in photosynthesis, it was decided to investigate the effective quantum yield and electronic transport rate in PS II in the *M. aeruginosa* cells treated with *E. equisetina* extract (Fig. 5). It was found that the effective quantum yields and the electronic transport rates were markedly decreased (eventually to zero) in the presence of the extracts at a dose of 10 µg or above. This revealed that the electronic transport from PS II to PS I was interrupted; the effective quantum yields in PS II reaction centers were limited, thereby leading to the disruption of photosynthesis and ensuing cell death.

### The Role of the Extract in Cell Kill

**Figure 6:**
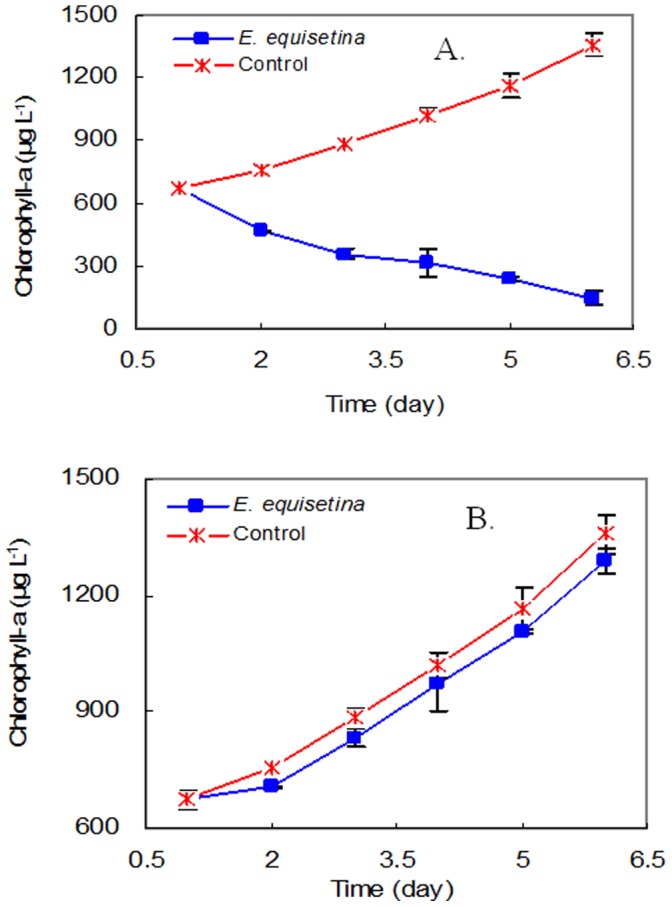
The growth of *M. aeruginosa* A) exposed to sterilized *E. equisetina* extract and B) pre-treated with strong illumination.

When the *M. aeruginosa*, in vitro, was exposed to the sterile, filtered (0.22 µm pore size) *E. equisetina* extract at a dose of 50 µg, the chlorophyll-a concentration was significantly decreased (Fig. 6A). This indicated that the inhibitory growth of *M. aeruginosa* was not due to microorganisms in the extract. In addition, when the *E. equisetina* extract was exposed to strong illumination (8000±200 Lux) at 35°C for seven days, the *M. aeruginosa* growth was not significantly different from that in the control (*p*>0.05) (Fig. 6B). These observations indicate that at least the constituents of the extract responsible for the cell-killing had degraded under strong illumination and elevated water temperature. Among the compounds identified in the aqueous *E. equisetina* root extract, flavonoids are quite readily photo-degraded [23]. This is noticeably so for those possessing an hydroxyl group attached to C-3 of ring C [24], [25].

The flavonoids are known to exhibit a wide range of biological effects including antibacterial and antiviral activities [26]. Indeed, the cyanobacterium (*M. aeruginosa*) is a kind of phytoplankton-bacteria. It is well-reported in the literature that flavonoids such as catechins, vanillin [27], and flavonol glycosides [28] are capable of inducing cyanobacterial death.

In summary, the results of this study provide strong evidence for the significant role that *E. equisetina* root extract, as an ensemble of active ingredients, plays in the induction of cyanobacterial death and control of cyanobacterial blooms as well as the benefits it provides to the aquatic ecosystem health. To our knowledge, this is the first reported use of a plant extract to remove cyanobacteria and/or algae both *in vitro* and *in situ* that has been characterized kinetically by exponential decay regression curves. Moreover, the use of environmentally benign active ingredients such as flavonoids extracted from *E. equisetina* root showed substantial “chemical attack” on cyanobacteria through destruction of the thylakoid membrane, interruption of electron transport, reduction of effective quantum yield, thus causing cessation of photosynthesis and thereby inducing the cyanobacterial death. Although we are still in the early stages of comprehending the exact responses of cyanobacteria and/algae to the likely synergistic actions of complex mixtures of compounds from natural materials in surface ecosystem management, this study opens the door to understand the dynamic behavior and the photosynthetic response.

## Materials and Methods

### Ethics Statement

Although this study involves in invertebrates, macrophyte species, or non-living materials, it did not include the use of non-human primates in research. The use of carps was approved by the local authority ethics committee (Shangshuangxiang). The location of the field studies is not protected in any way. No specific permits were required for the described field studies. Moreover, the field study involving invertebrate, macrophyte species did not involve endangered or protected species. During the field study, the survey of invertebrate, macrophyte species was known and permitted by the local authority.

### Preparation of Extracts for Laboratory Experiments

Aqueous *E. equisetina* root extract was prepared by adding 3.0 g air-dried root (chopped into 0.5 cm lengths) to 210 mL reverse-osmosis (RO)-purified water in a new lidded gallipot at 70±5°C for 2 h. The prepared extract was stored in a refrigerator (∼2−4°C) for the experiments.

### Preparation of Extract for Field Trials

The preparation of the *E. equisetina* extract for the field trials was simply a scaled up version of that used for the indoor laboratory experiments with the herb:water ratio  = 1∶70 w/v. The extract for field trials was applied at a dose of 87.5 mL/m^3^ water (equivalent to 1.25 g dried *E. equisetina* root per m^3^ water).

### Laboratory Bioassays

*M. aeruginosa* was selected for the bioassays, which were performed in triplicate. The sterilized water used for cyanobacterial cultivation was prepared according to the method of Jin and Dong with a pH 7.5 and salinity 30‰ [29]. The organism was grown in BG11 medium [30] at 25±1°C in incubators with illumination at 3,000 Lux over a 12 hr light:12 hr dark cycle. All flasks containing the cyanobacteria were placed on a shaker (150 rpm). The *M. aeruginosa* was cultured to the log growth phase. For the controls, 1.0 mL of *M. aeruginosa* culture was placed into 250 mL sterilized flasks containing fresh BG-11 medium and the total volume was brought to 200 mL with the cell density such that the concentration of chlorophyll-a measured ∼670 µg/L.

Bioassays were performed by the addition of *E. equisetina* extract (doses  = 10 µg, 25 µg, 50 µg and 75 µg) to separate flasks, each with an initial population of *M. aeruginosa* as measured by chlorophyll-a (∼670 µg L^−1^), and then incubated at the same conditions as those in the culture of *M. aeruginosa* mentioned above.

To test whether other microorganisms could have caused the growth inhibition of *M. aeruginosa*, the sterilized extract (passed through 0.22 µm pore size, dose  = 50 µg) of *E. equisetina* was added to BG11 medium with *M. aeruginosa* under ultrasterile conditions with the bioassay being performed as above.

To test the effect of illumination and thermalization on its inhibition properties, 250 mL glass bottles with unfiltered extract of *E. equisetina* were placed in an incubator with strong illumination (8000±200Lux) and high temperature (35±1°C) for seven days and then the bioassay was performed as above.

To determine the effect of the crude extract on photosynthesis of cyanobacterial cells, a series of unfiltered extracts of different concentrations were added to flasks containing BG-11 medium. Cyanobacteria cultured at log phase were inoculated into the media in triplicate, each in a 150 mL container with 100 mL of medium at 28±1°C at light intensity of 2,500 Lux under a 12 hr light:12 hr dark cycle. The initial cell density of cyanobacteria corresponded to chlorophyll-a concentration of ∼670 µg L^−1^. Samples (0.5 mL) of suspension were withdrawn for measurement of the effective quantum yields and electronic transport rates.

### Application of E. Equisetina Extract in the Field

The extract of *E. equisetina* was applied to six ponds in Kunming, Western China from April to June, 2008. The area of each pond was between ∼1000−1200 m^2^ with an average depth of 1.2 m. These ponds were connected to each other before the experiment, to imply they had similar conditions including hydrology, water quality and biological composition. These ponds were eutrophic, experiencing frequent cyanobacterial blooms and high nutrient levels (n = 6, total nitrogen  = 3.50 mg L^−1^ to 12.60 mg L^−1^ and phosphorus  = 0.34 mg L^−1^ to 1.56 mg L^−1^). Water samples (in triplicate) were collected from each pond at a depth of 25 cm below the surface, approximately 2 m from the shore, and analyzed for chlorophyll-a to gauge bacterial cell density.

### Fish Status Experiment

Each pond was stocked with 1000 fish (Crucian carp, Grass carp, and Silver carp, in proportions of 2∶1:1) two months prior to commencing the experiment. The feeding of the fish in each pond was the same throughout the experimental period. At the beginning of the experiment, the ponds were separated, and the treated pond received an application of the *E. equisetina* extract (with the dose being equivalent to 1.25 g *E. equisetina* root/m^3^ of water). No further extract was added during the experimental period. For the control pond, no *E. equisetina* extract was added during the experimental period. Fish death was determined by counting the number of dead fish floating on the water surface. The fish survival number was the difference between the total fish and the dead ones. The fish survival rate was the ratio of fish survival numbers to the total fish number. The fish yield was the fresh weight (in three times when the fish was harvested at the end of the experiment.

### Chemical and Spectroscopic Analyses

Total nitrogen (TN), total phosphorus (TP), dissolved total phosphorus (TDP), nitrate (NO_3_-N) and ammonium (NH_4_-N) nitrogen were determined using the standard methods described by APHA [31]. The water samples were filtered (pore size 0.45 µm) for chlorophyll-a analysis. All filters were immediately placed on ice and transferred to a freezer at −20°C until analysis (< one month). Chlorophyll-a was measured at 663 nm and 750 nm after overnight acetone (90% v/v) extraction of the filters [32].

The effective quantum yields and electronic transport rates of photosystem II (PS II) in cyanobacterial cells were determined with a Phyto-PAM fluorimeter (Waltz, Germany) by using the saturating pulse method [33].

### Field Measures of Ecological Health

The macrophytic vegetation associated with the experimental lakes was sampled using transects. Details of the method have been previously described [34]. Zooplankton samples were collected from the water 0.5 m below the surface. Ten liters of water per sample were filtered through a plankton net (20 µm mesh). Each sample of net-residue was immediately preserved in 4% formalin buffered with borate. Simpson’s diversity index [35] was used to quantify the biodiversity of macrophytes and zooplankton.

Characterization of bacteria carrying the flaA gene from natural biofilms in the ponds was conducted using the ERIC-PCR technique [36]. The forward oligonucleotide primer used was (5′-ATGTAAGCTCCTGGGGA-TTCAC-3′) with the reverse primer being (5′AAGTAAGTGACTGGGG-TGAGCG-3′) [37]. Freeze-dried natural biofilm (1.0 g) was centrifuged for 10 min, with DNA being extracted from periphyton by the method of Di Giovanni *et al* [38]. PCR amplification of the flaA gene was performed using the forward primer (5′-AGCTCTTAGCTCCATGAGTT-3′) and the reverse primer (5′-ACATTGTAGCTAAGGCGACT-3′) [39]. Reactions were performed in 50 µL volumes using a Perkin Elmer thermocycler (Gene Amp PCR System 2,400). Reaction mixtures were 1 µM in each primer, 50 to 100 ng of genomic DNA, 1.5 mM MgCl_2_, with each deoxynucleoside triphosphate (dATP, dTTP, dCTP, dGTP; Promega, USA) at a concentration of 0.2 mM, and 1 U of Taq DNA polymerase (Promega, USA). For flaA amplification, cycle conditions of 1 cycle at 94°C for 2 min; 35 cycles at 94°C for 1 min, 65°C for 30 s, and 72°C for 30 s; and 1 cycle at 72°C for 10 min were used. For ERIC-PCR, the amplification was accomplished by running 30 cycles of denaturing at 90°C for 30 s, annealing at 50°C for 30 s and extending at 50°C for 30 s, and the initial denaturing at 95°C for 5 min, initial extending at 72°C for 8 min. All amplification products were electrophoresized in agarose gels, stained with ethidium bromide, detected under a short-wavelength UV light source, and photographed with a Polaroid 667 camera. The 100 bp DNA Ladder (Promega) was used as a molecular size marker. Shannon-Weaver diversity index [40] of bacteria carrying the flaA gene in natural biofilms was calculated based on the relative area of the ERIC-PCR fingerprint peak.

An exponential decay curve [Eq (1)] was employed to investigate the kinetics of the reduction of cyanobacteria (*M. aeruginosa*) in the presence of *E. equisetina* extract.


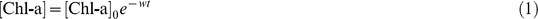


Where, [Chl-a] is the chlorophyll-a concentration of cyanobacteria at time t. [Chl-a]_0_ is the initial chlorophyll-a concentration of cyanobacteria. *w* is the first-order rate constant (day^−1^), and t is the time since extract addition (day).

All samples were collected in triplicate. Significant differences between control and treated samples were determined using one-way ANOVA (SPSS version 17.0). The level of statistical significance was accepted when *p*<0.05.
